# Trajectory and predictors of cancer- and treatment-related fatigue (CRF) in adolescent and young adult cancer patients: a prospective, longitudinal study

**DOI:** 10.1186/s12885-026-16324-4

**Published:** 2026-06-16

**Authors:** Alexandre Chan, Daniella Chan, Parisa Agrawal, Lucas Lee Beppu, Khai Kober, Ding Quan Ng, Michael Sayer, Julia Trudeau

**Affiliations:** 1 Department of Clinical Pharmacy Practice, University of California Irvine, 802 W Peltason Dr, Irvine, CA 92697-4625 USA; 2 Department of Oncology Pharmacy, National Cancer Centre Singapore, Singapore, Singapore

**Keywords:** Adolescent and young adult cancer patients, Cancer, Cancer-related fatigue, Cytokines, Fatigue

## Abstract

**Background:**

Although cancer- and treatment-related fatigue (CRF) is well recognized as a clinically important and distressing symptom among adolescent and young adult cancer (AYAC) patients, the understanding of the underlying mechanisms behind CRF in AYAC remains limited. We evaluated the trajectory and associated predictors of CRF among AYAC patients (Clinicaltrials.gov: NCT03476070, registered 03/23/2018).

**Methods:**

Newly diagnosed AYAC (15–39 years old) and non-cancer controls (NC) recruited from National Cancer Centre Singapore completed clinical assessments and provided blood draws for inflammatory cytokine measurements every 3 months up to 12 months. Mixed-effects models were used to evaluate significant factors associated with fatigue (MFSI-SF Total score), with AYAC stratified by chemotherapy status (active chemotherapy vs. >30 days post-chemotherapy).

**Results:**

Fifty AYAC and 105 NC participants were included, with nearly 1 in 3 AYAC participants reported clinically important CRF at some point. Measurements collected during active chemotherapy had a higher rate of clinically important CRF symptoms relative to samples collected post-chemotherapy and from non-cancer controls. Regression analyses evaluating significant influencers of fatigue scores demonstrated that active chemotherapy, female sex, worsened psychological distress scores, and increased IL-10 levels were associated with worse self-reported fatigue symptoms (all *p* < 0.05).

**Conclusion:**

Given the observed prevalence of clinically important CRF amongst AYAC within our study, our results further demonstrate that these patients are vulnerable to fatigue challenges. Identifying factors associated with these debilitating symptoms enables clinicians to better recognize at-risk patients and proactively address symptom burden.

**Supplementary Information:**

The online version contains supplementary material available at 10.1186/s12885-026-16324-4.

## Background

Cancer- and treatment-related fatigue (CRF) refer to a distressing, persistent, subjective sense of physical, emotional, and/or cognitive tiredness related to the disease or anticancer treatments [1]. Notably, patients rate fatigue as being more distressing than other cancer- or treatment-induced symptoms, having significant consequences on cognitive function and emotional well-being [1, 2]. These CRF-related challenges can often augment neuropsychiatric complications, underscoring their utmost importance in the management of patient well-being.

The occurrence of fatigue is highly prevalent among cancer patients and survivors. Studies have shown that more than 90% of patients who receive chemotherapy or radiotherapy experience CRF [3–5]. CRF is acknowledged as a clinically relevant side effect among adolescent and young adult cancer (AYAC) patients; In our previous study, we found that 36.9% newly diagnosed AYAC patients experience CRF one month after diagnosis, with the incidence reaching 39.0% in patients between 24 and 39 years old [6]. As one of the most distressing and severe symptoms experienced by AYAC patients, CRF has been found to hinder regular activities and attendance to school and work [7, 8]. Many known risk factors of CRF identified amongst AYAC patients can be attributed to treatment related toxicities, such as certain types of anemia, hormone dysregulation, and sleep distrubances [9]. Others suggested that socioeconomic factors can play a role, such as nutrition status, financial stressors or routine medical access [10]. Clearly, CRF is an unmet need in AYAC survivorship and optimal strategies to manage CRF are urgently needed at this time.

The underlying etiology of CRF remain unclear. The presence of cancer and treatment toxicities cause significant stress, leading dysregulated systemic processes, and fatigue symptoms. Concurrent neuropsychiatric symptom burden may further play a role, including depressive disorders, anxiety, and altered sleep patterns [11–13]. A hypothesized biological basis for CRF is cytokine signaling dysregulation, which can result from inflammatory responses to chemotherapeutic agents or cytokine release in the tumor microenvironment [14–16]. Cytokines can cause disruptions in the blood-brain barrier, as well as structural and cellular changes in the central nervous system, which have been associated with fatigue [17]. Characterizing a concise trajectory of the onset of fatigue symptoms is challenging because there is significant variance with respect to the physiological response to cancer and cancer treatment. However, studies emphasizing symptom onset during cytotoxic treatments have suggested peak reported fatigue symptoms occurred ~ 8 weeks from treatment initiation, and again upon completion of chemotherapy [18]. While there are several different speculated risk factors for CRF, few studies have examined patient characteristics, treatment information, circulating biomarker data, and concurrent symptom burdens to better understand risk factors for CRF.

In this study, we leveraged the Adolescent and Young Adult Cancer Patients: Cognitive Toxicity on Survivorship (ACTS) study to understand the extent of fatigue symptoms experienced by a diverse cohort of AYAC patients [19]. Unlike in pediatric and older cancer populations, AYAC patients are significantly understudied, with limited prospective data evaluating long-term trajectory and predictors of CRF. Improving our understanding will be key to delivering timely support as they balance cancer survivorship with major life milestones like higher education, career development, and family planning. Hence, the primary objective of this study was to evaluate the trajectory of CRF among newly diagnosed AYAC patients stratified by chemotherapy status. The secondary objective involved an evaluation of the predictors (including clinical characteristics and plasma biomarkers) associated with CRF in AYAC patients.

## Methods

### Study design

This is a secondary analysis of the ACTS study [19], a multicenter, prospective, longitudinal, observational study conducted in Singapore between June 2018 and June 2022 to evaluate cognitive toxicities in AYAC patients (Clinicaltrials.gov: NCT03476070, registered 03/23/2018). ACTS was conducted at the National University of Singapore, National Cancer Centre Singapore, and KK Women’s and Children’s Hospital between June 2018 and December 2021. Over 70% of all AYAC in Singapore receive ambulatory cancer care from NCCS and KKH. Candidates were screened by research staff within these institutions and subsequently approached to participate in the study if they met eligibility criteria. The study protocol received ethics approval from the SingHealth Institutional Review Board (CIRB 2017/3139) and all study participants provided written informed consent prior to participation.

### Participant eligibility criteria

AYAC and non-cancer community controls (NC) were recruited. AYAC participants were between 15 and 39 years of age, newly diagnosed with cancer (within 1 month), treatment naïve (surgery, chemotherapy, radiotherapy), scheduled to receive chemotherapy, and were able to give informed consent (and parental consent if required). NC were age-matched to AYAC within three years, meeting the same eligibility criteria (except cancer-specific elements). For both groups, those with evidence of psychosis or underlying neuropsychiatric illness that might impair cognitive abilities were excluded. Additionally for NC, those with immediate family members enrolled as AYAC in the study were excluded.

### Procedures

Data collection at each time point occurred with each study visit, where subjects would provide peripheral blood samples with concurrent clinical assessments. All AYAC were evaluated at baseline prior to receiving anti-cancer treatment (T1). Four subsequent study visits were scheduled in 3-month intervals (T2, T3, T4, T5) spanning 1 year. Age-matched NC were evaluated at baseline (T1) and six months (T3) post-baseline. Amongst AYAC patients, participation varied from 1 to 4 post-baseline study visits at varying timepoints, while all NC patients provided 1 post-baseline sample.

### Clinical measurements

The primary outcome of this study was fatigue symptoms, represented by cumulative fatigue score based on survey responses from the Multidimensional Fatigue Symptom Inventory-Short Form (MFSI-SF) questionnaire [20, 21]. The survey contains 30 distinct items representative of different fatigue symptoms, where patients rank the extent of which they are experiencing them on a Likert scale of 0 to 4. Summed responses represent cumulative fatigue score, with higher scores indicating worsening symptoms. Both English and Chinese versions of the MFSI-SF were validated in Singapore [20, 22, 23].

As a secondary outcome, we reported the percentage of participants who developed clinically important fatigue symptoms, defined as a ≥ 10.79-point increase in MFSI-SF total score from baseline. This threshold represents the minimally clinically important difference (MCID) that was previously established as a conservative, empirically validated cutoff in breast cancer survivors [24]. These percentages were reported cumulatively across all entries for AYAC and NC patients, in addition to different sub-groups of measurements based on collection timepoint or chemotherapy treatment status. Currently, there is no standard clinical threshold within the MFSI scale that designates “significant” symptoms based on a cross-sectional score; reporting score change via the MCID represents the most appropriate means of capturing clinically meaningful symptoms .

Additional clinical assessment tools were utilized in the study to further characterize participants’ symptomatic burden and health, including the Rotterdam Symptom Checklist (RSCL) psychological distress subscale [25, 26] and the Pediatric Quality of Life Inventory (PedsQL) emotional functioning subscale [27, 28]. These surveys contain Likert scale questions summed to create scores describing psychological distress and emotional functioning.

Additional details of these assessments can be found in Supplementary Methods.

### Circulating cytokines levels

To measure circulating inflammatory cytokines, a 9-ml blood sample was collected at each timepoint. Samples were stored in ethylenediaminetetraacetic acid tubes, and centrifuged at 1069 x *g* for 10 min at 4*°*C. The plasma was stored in aliquots in a − 80*°*C freezer prior to analysis. Plasma levels of cytokines (pg/mL) interferon (IFN)-ƴ, tumor-necrosis factor (TNF-α), granulocyte-macrophage colony-stimulating factor (GM-CSF), and interleukin (IL)-2, IL-4, IL-6, IL-8, and IL-10, were analyzed using the multiplexed immunoassay (Bioplex Human Cytokine 9-Plex Panel, Biorad). Biomarkers with more than 80% of values below the lower limit of quantification were excluded from the final analysis [29]. For analysis purposes, biomarker values were logarithmically transformed after being increased by 0.1.

### Longitudinal sampling

Given the longitudinal study design, samples were labeled specific to the timepoint at which they were collected (T1-T5). To further characterize AYAC cancer treatment status over time, data from post-baseline visits were labeled as occurring during active chemotherapy (within 30 days of chemotherapy administration) and post-chemotherapy (> 30 days after last dose). In addition to inclusion/Exclusion criteria at the patient level, collected samples with concurrent clinical assessments were only if they met “complete case” criteria. Complete cases entailed each observation having no missing clinical assessments or biomarkers measurements.

### Baseline characteristics

Additional collected data included patient age at study initiation, biological sex, race/ethnicity, years of education received, and primary cancer diagnosis. Patient age and years of education were treated as numeric variables. Other features are included in descriptive tables and are not included in statistical modeling efforts, due to sample size constraints or lack of direct relevance to study outcomes.

### Statistical comparisons

Initially, characteristics between AYAC and NC cohorts were compared using Welch’s t-test or Mann-Whitney U test for continuous variables, and Chi-square test or Fisher’s exact test for categorical variables. To characterize the prevalence of significant fatigue symptoms, the total unique instances of clinically important fatigue are reported for all included complete cases. Additionally, the number of subjects experiencing clinically important fatigue symptoms at any point is reported.

### Preliminary regression analyses

For initial regression analyses, we separated AYAC and NC participants into separate cohorts. Using MFSI-SF Total score as the primary outcome, evaluated the impact of treatment status (active chemotherapy, post-chemotherapy, ref= baseline measurements) as the primary variable of interest, adjusted for patient baseline characteristics (age, sex, race/ethnicity, years of education). Amongst NC patients, sample timing (T3 measures, ref=baseline measurements) was the primary variable of interest and was adjusted for the same characteristics. As an exploratory measure, we repeated this analysis utilizing secondary clinical outcome measures (RSCL Psychological Distress, PedsQL Emotional Functioning) and measured cytokine levels as separate outcomes.

To further explore cytokine level trends, we conducted this analysis again amongst 4 distinct cohorts stratified by cancer status and onset of clinically important fatigue symptoms. Patients with all associated samples/assessments were separated into one of the following groups: AYAC patients who experienced clinically important fatigue, AYAC who did not, NC who experienced clinically important fatigue, and NC patients who did not.

### Stepwise model building predicting cumulative fatigue score (MFSI-SF)

Finally, to characterize how biomarkers influence fatigue symptoms, stepwise multivariate regression analysis was implemented with fatigue score (MFSI-SF Total score) as the outcome, with random intercepts accounting for within-subject correlation. A structured four-stage model-building process was implemented, with each stage evaluating a distinct class of variables. At each stage, model selection was guided by Akaike Information Criterion (AIC) to optimize model fit, starting with all potential predictors. Model stability was indicated by lower AIC values. Features having the highest p-value (based on Wald-Test Statistic) were removed in a stepwise fashion, and AIC values were subsequently evaluated. Feature reduction stopped when the removal of an additional variable led to an increased AIC value. Optimized models from each stage were carried forward into the next stage, where a new class of potential predictors was evaluated. Features selected in optimized models in earlier stages were not eligible for removal in subsequent stages. Data from baseline assessments (Timepoint 1) were not included as unique entries, it was utilized to characterize change relative to baseline for several characteristics (post-baseline measure – baseline measure) and baseline fatigue symptoms.

Model stages were characterized by classes of variables considered in each step, which included: chemotherapy status and baseline fatigue score (Stage 1), patient demographic characteristics (Stage 2), concurrent clinical symptoms (Stage 3), and measured cytokine levels (Stage 4). Change in PROs and biomarker levels relative to baseline (post-baseline measure – baseline measure) was used as predictors for stages 3 and 4, respectively. Model building statistics, including cumulative AIC values, regression coefficients, coefficients of determination, and Wald-statistics for individual features, are reported. After conducting this analysis amongst all participants, a sub-analysis was done utilizing AYAC samples only.

All statistical tests were two-sided, and a P-value < 0.05 was considered statistically significant. Statistical analyses were performed using R version 4.4.1.

## Results

### Eligible patients and baseline patient characteristics

We initially approached 115 total AYAC patients to participate in the study, of which 75 agreed to study participation [19]. For this secondary analysis, only patients having needed data from biospecimens with concurrent symptom measurements were eligible for inclusion (complete case analysis), leaving 50 AYAC and 105 NC participants included in the study. AYAC (mean = 32.7, SD = 5.6) and NC (mean = 31.1, SD = 4.8) had comparable ages (Table 1), with some significant racial/ethnic differences between the groups (*P* = 0.0025) (Table 1). Socioeconomic background also varied, with AYAC having fewer mean years of education [mean (SD): 14.9 (3.6) vs. 17.5 (3.2), *P* < 0.001] compared to NC (Table 1). AYAC patients represented a diverse, heterogeneous cancer patient population including nasopharyngeal cancer (NPC) (24.0%), gynecological cancers (22.0%), lymphoma (16.0%), and breast cancer (16.0%). Treatment exposures were also variable, with 50% of patients received concurrent radiotherapy (50.0%), and 36% had treatment-related surgery (Table 1).

**Table 1:** Baseline Characteristics Amongst Adult and Young Adult Cancer Patients and Non-Cancer Control Participants

	AYAC (*N* = 50)	NC (*N* = 105)	^a^*P*-value
Age, mean (SD)	32.7 (5.6)	31.1 (4.8)	0.081
Gender, N (%)			
Male	21 (42.0)	36 (34.3)	0.45
Female	29 (58.0)	69 (65.7)	
Ethnicity, N (%)			
Chinese	38 (76.0)	79 (75.2)	0.0025**
Malay	7 (14.0)	2 (1.9)	
Indian	2 (4.0)	19 (18.1)	
Others^b^	3 (6.0)	5 (4.8)	
Years of Education, mean (SD)	14.9 (3.6)	17.5 (3.2)	< 0.001***
Cancer-specific Characteristics			
Cancer Type, N (%)			
NPC	12 (24.0)	NA	NA
Gynecological	11 (22.0)	NA	NA
Lymphoma	8 (16.0)	NA	NA
Breast	8 (16.0)	NA	NA
Others^c^	11 (22.0)	NA	NA
Treatment Type, N (%)			
Chemotherapy	49 (98.0)	NA	NA
Radiotherapy	25 (50.0)	NA	NA
Surgery	18 (36.0)	NA	NA
Hormonal	3 (6.0)	NA	NA
Immunotherapy	2 (4.0)	NA	NA
Assessments			
^d^MFSI-SF, mean (SD)			
General Subscale	5.3 (4.1)	4.8 (4.5)	0.49
Physical Subscale	3.1 (3.6)	2.2 (3.0)	0.12
Emotional Subscale	6.5 (4.5)	3.1 (3.5)	< 0.001***
Mental Subscale	2.8 (3.6)	3.1 (3.5)	0.57
Vigor Subscale	13.3 (4.7)	15.1 (5.0)	0.029*
Total Score	4.4 (16.4)	-1.9 (15.6)	0.026*
^e^RSCL, mean (SD)			
Physical Symptom Distress	12.8 (12.2)	8.3 (7.4)	0.017*
Psychological Distress	28.5 (19.1)	16.3 (15.3)	< 0.001***
Activity Level	4.7 (7.8)	0.06 (0.4)	< 0.001***
Overall Valuation of Life	23.6 (18.5)	16.0 (16.2)	0.017*
^f^PedsQL, mean (SD)			
Physical Functioning	79.9 (20.4)	92.3 (11.5)	< 0.001***
Emotional Functioning	66.2 (20.4)	78.9 (19.5)	< 0.001***
Social Functioning	90.3 (14.0)	91.2 (12.4)	0.70
Work/School Functioning	79.6 (18.1)	85.6 (15.0)	0.052
Cytokines, median (IQR)			
IL-2 (pg/mL)	0.0 (0.0, 1.3)	0.0 (0.0, 0.0)	0.0043**
IL-4 (pg/mL)	0.0 (0.0, 0.6)	0.0 (0.0, 0.0)	0.0018**
IL-6 (pg/mL)	2.0 (1.0, 3.3)	0.5 (0.0, 1.0)	< 0.001***
IL-8 (pg/mL)	5.5 (3.5, 11.9)	4.3 (2.5, 5.6)	< 0.001***
IL-10 (pg/mL)	0.5 (0.0, 1.6)	0.0 (0.0, 0.5)	0.0036**
IFN-ƴ (pg/mL)	0.8 (0.3, 1.6)	0.3 (0.0, 0.6)	< 0.001***
TNF-α (pg/mL)	9.7 (6.2, 15.7)	9.2 (6.5, 12.0)	0.39

*Abbreviations*: *AYAC* adolescent and young adult cancer, *IFN-ƴ* interferon-gamma, *IL* interleukin, *IQR* interquartile range, *MFSI-SF* Multidimensional Fatigue Symptom Inventory – Short Form, *NA* not applicable, *NC* non-cancer control, *NPC* nasopharyngeal cancer, *PedsQL* Pediatric Quality of Life Inventory, *RSCL* Rotterdam Symptom Checklist, *SD* standard deviation, *TNF-α* tumor necrosis factor-alpha

*P < 0.05, **P < 0.01, ***P < 0.001

^a^P-values for continuous variables were obtained using t-test or Mann-Whitney U test, and for categorical variables using Chi-square test or Fisher’s exact test

^b^Other ethnicities include Filipino (*N* = 4) and Vietnamese (*N* = 1) among NC, as well as Bhutanese (*N* = 1), Filipino (*N* = 1), and Sikh (*N* = 1) among AYAC

^c^Other cancer types include: Testicular (*N* = 5), Colorectal (*N* = 2), Gastrointestinal Stoma (*N* = 1), Lung (*N* = 1), Mandibular (*N* = 1), and Sarcoma (*N* = 1)

^d^Higher MFSI-SF subscale scores, except for Vigor, indicate worse fatigue symptoms; higher MFSI-SF score indicates worse fatigue symptoms

^e^Higher RSCL subscale and overall scores represent greater physical and psychological symptom distress

^f^Lower PedsQL subscale scores represent worse physical and psychosocial functioning

At baseline, AYAC patients had significantly higher fatigue symptoms relative to NC (MFSI-SF Total Score, *P* = 0.026) (Table 1). AYAC patients also had significantly lower quality of life scores (RSCL Overall Valuation of Life, *p* = 0.01), and more impaired physical (PedsQL Physical Functioning, *p* < 0.001) and emotional function (PedsQL Emotional Function, *p* < 0.001) relative to NC. AYAC had significantly higher baseline plasma levels of several cytokines, including IL-2, IL-4, IL-6, IL-8, IL-10, and IFN-ƴ (all *p* < 0.05) (Table 1). GMCSF was removed from analysis as > 80% of the samples had undetectable plasma levels.

### Study participation and sampling over time

A total of 383 complete cases were included amongst 50 AYAC and 105 NC participants (Supplemental Table 1). For AYAC, 123 total complete cases were utilized from post-baseline assessments, with all participants having baseline (T1) data. The total observations by timepoint varied, with 35 occurring during active chemotherapy and 88 taken post-chemotherapy. AYAC study participation varied, with participants having anywhere from 2 to 5 total observations included (Supplemental Table 1). Amongst NC, all 105 participants provided baseline (T1) and timepoint 3 (T3, ~ 6 months from baseline) observations.

### Clinical symptom and cytokine measures over time

With post-baseline reporting, a higher percentage of AYAC patients experienced clinically important fatigue symptoms at some point in the study (30.0%, *n* = 15) relative to NC (21.0%, *n* = 22) (Table 2). Amongst complete cases collected, 59.3% (*n* = 73) amongst AYAC reported clinically important fatigue symptoms compared to 21% (*n* = 22) amongst NC participants. The incidence of clinically important fatigue symptoms was higher amongst observations during Active Chemotherapy (*n* = 31, 88.6%), compared to post-chemotherapy (*n* = 42, 47.7%) (Table 2). Considering cumulative MFSI-SF score, mean score significantly varied across timepoints amongst AYAC patients (Supplemental Table 2). Mean values grouped by chemotherapy status showed patients receiving active chemotherapy (mean = 7.9, SD = 18.0) having higher fatigue scores compared to post-chemotherapy (mean= -2.4, SD = 16.0) (Supplemental Table 3). Mean fatigue score for post-baseline observations (mean= -0.1, SD = 16.7) for NC participants varied (Supplemental Table 3). Trends with respect to other clinical measures can be further observed in Supplemental Tables 2 and 3. Considering cytokine levels, measures were highly variable over time and amongst study groupings for all cytokines assessed (Supplemental Tables 2 and 3).

**Table 2:** Incidence of Clinically Important Fatigue Symptoms Amongst Participants and Complete Cases Collected

	NC (*n*,%)	AYAC (*n*,%)
Individuals Experiencing Clinically Important Fatigue Symptoms NC = 105 participants AYAC = 50 participants	22 (21.0)	15 (30.0)
Post-Baseline NC Samples (*n* = 105 samples)	22 (21.0)	-
Active chemotherapy (*n* = 35 samples)	-	31 (88.6)
Post-chemotherapy (*n* = 88 total samples)	-	42 (47.7)

### Preliminary regression results predicting clinical measures and cytokine levels

Chemotherapy status significantly measured fatigue symptoms (MFSI-SF Total Score) amongst AYAC patients in the study. (Active Chemotherapy- Coef (95% CI): 0.7 (-4.0, 5.5), Std Coef = 0.04, *p* = 0.78; Post Chemotherapy- Coef(95% CI):-6.3 (-10.1, -2.5), Std Coef= -0.37, *p* = 0.0015; ref= Baseline Measurements). Amongst secondary clinically outcomes it similarly influenced symptom burden, as measured by RSCL Psychological Distress Score (Active Chemotherapy- Coef (95% CI): -5.1 (-10.4, 0.4), Std Coef= -0.28, *p* = 0.072; Post Chemotherapy- Coef (95% CI): -10.9 (-15.3, -6.5), Std Coef=-0.61, *p* < 0.001; ref= Baseline Measurements), and PedsQL Emotional Function (Active Chemotherapy- Coef (95% CI): 2.5 (-3.8, 8.6), Std Coef = 0.12, *p* = 0.43; Post Chemotherapy- Coef (95% CI): 12.6 (7.7, 17.6), Std Coef = 0.62, *p* < 0.001, ref= Baseline Measurements) (Table 3). With all results, post-chemotherapy observations were associated with significantly less symptomatic burden or improved function relative to baseline measures (Table 3). For NC participants, only MFSI-SF Total Score was significantly influenced by measurement timing (Coef (95% CI): 1.7 (0.4, 3.1), Std Coef = 0.10, *p* = 0.012; ref= Baseline Measurements) (Table 3).

**Table 3:** Impact of Treatment Status on Cumulative Symptom Scores Based on Preliminary Regression Analysis Amongst Adolescent and Young Adult Cancer Patients and Non-Cancer Controls

(A) AYAC		
Assessments	Active Chemotherapy *ref= Baseline Measurements*	Post Chemotherapy *ref= Baseline Measurements*
MFSI-SF Total	B=0.7, 95% CI (-4.0, 5.5)	B=-6.3, 95% CI (-10.1, -2.5)
	β=0.04	β=-0.37
	p= 0.78	p= 0.0015**
RSCL Psychological Distress	B=-5.1, 95% CI (-10.4, 0.4)	B=-10.9, 95% CI (-15.3, -6.5)
	β=-0.28	β=-0.61
	p= 0.072	P<0.001***
PedsQL Emotional Functioning	B=2.5, 95% CI (-3.8, 8.6)	B=12.6, 95% CI (7.7, 17.6)
	β=0.12	β=0.62
	p= 0.43	P<0.001***

(B) NC		
**Assessments**	**Post-Baseline ** *ref= Baseline Measurements*	
MFSI-SF Total	B=1.7, 95% CI (0.4, 3.1)	
	β=0.10	
	p= 0.012*	
RSCL Psychological Distress	B=0.9, 95% CI (-0.6, 2.4)	
	β=0.05	
	p= 0.26	
PedsQL Emotional Functioning	B=0.1, 95% CI (-1.5, 1.7)	
	β=6.01E-3	
	p= 0.88	

The first column represents different symptom scores utilized as outcomes for regression analysis, with subsequent columns representing treatment status (for adolescent and young adult samples (AYAC)) or post-baseline measurements (for non-cancer controls (NC)). Reported results are regression coefficients (B), 95% confidence intervals ), followed by standardized regression coefficients (β), and their corresponding Wald statistic reported below them. For MFSI-SF Total and RSCL Psychological Distress Score outcomes, positive coefficients suggest a given treatment phase/post-baseline assessment is associated with increased symptom scores (worse symptom burden) relative to baseline measures. For PedsQL Emotional Functioning, negative coefficients suggest a given treatment phase/post-baseline assessment is associated with decreasing emotional function score (worse emotional functioning) relative to baseline measures. Panel (**A**) represents regression results amongst AYAC participants, while panel (**B**) represents regression results for non-cancer controls

*P<0.05,**P<0.01, ***P<0.001

Amongst all AYAC participants, chemotherapy status did not significantly influence cytokine levels (Supplemental Table 4, Panel A). With AYAC participants separated by occurrence of clinically important fatigue symptoms, chemotherapy status still had no significant influence on measured cytokine levels for either group (Supplemental Table 5, Panels A & B). Unexpectedly, several post-baseline cytokine levels amongst NC participants were significantly influenced by measurement timing, including amongst all NC participants (Supplemental Table 4, Panel B) and amongst sub-cohorts separated by onset of clinically important fatigue symptoms (Supplemental Table 5, Panel C & D).

### Stepwise model building results identifying significant predictors of cumulative fatigue score

With modeling efforts including all participants, participant age, education years, and change in PedsQL score were not included in the final model (Table 4, Supplemental Table 6). Chemotherapy status (Active Chemotherapy- Coef(95% CI): 4.22(0.13, 8.33), Std Coef = 0.24, *p* = 0.049; Post-Chemotherapy- Coef(95% CI): 3.86(-0.29, 7.87), Std Coef= -0.0014, *p* = 0.053) was significantly associated with cumulative fatigue score, suggesting a significant elevation in scores during active chemotherapy relative to assessments from non-cancer controls. Baseline fatigue measures (Coef(95% CI): 0.78(0.68, 0.88), Std Coef = 0.76, *p* < 0.001, numeric), race/ethnicity (Malay- Coef(95%): -9.61(-16.21, -3.03), Std Coef= -0.55, *p* = 0.006; Indian- Coef(95% CI): 2.88(-1.82, 7.60), Std Coef = 0.17, *p* = 0.24; Other- Coef(95% CI): 2.30(-5.06, 9.67), Std Coef = 0.13, *p* = 0.55; reference= Chinese), and Change in RSCL score (Coef(95% CI): 0.52(0.43, 0.60), Std Coef = 0.54, *p* < 0.001, numeric) were significant predictors of cumulative fatigue score as well. Amongst cytokines evaluated, both IL-4 and IL-10 were selected for inclusion in the final model, with only change in IL-10 levels significantly associated with cumulative fatigue symptoms (Coef(95% CI): 1.87(0.44, 3.30), Std Coef = 0.13, *p* = 0.013, numeric).

**Table 4:** Results Stepwise Regression Analyses Predicting Fatigue Score (MFSI-SF)

Category	Variable	AYAC + NC^a^ AIC = 1688.6	AYAC Only^b^ AIC = 919.1
Sample Timing (Relative to Chemotherapy) *Ref= Normal Control Samples (AYAC + NC)*	Active Chemotherapy	B = 4.22, 95% CI (0.13, 8.33), β = 0.24 *p* = 0.049*	B = 3.86, 95% CI (0.29, 7.87), β=-0.23 *p* = 0.053
*^Ref= Post Chemotherapy Samples (AYAC Only)*	Post Chemotherapy	B=-0.025, 95% CI (-3.58, 3.52), β=--0.0014 *p* = 0.99	*Reference Value^*
Baseline Fatigue Score (T1 measures) *(numeric* ***)***	MFSI-SF Total Score	B = 0.78, 95% CI (0.68, 0.88), β = 0.76 *p* < 0.001***	B = 0.77, 95% CI (0.58, 0.95), β = 0.78 *p* < 0.001***
Age *(numeric)*	Age (in years)		B = 0.19, 95% CI (-0.35, 0.70), β = 0.06 *p* = 0.52
Gender *Ref= Male (binary variable)*	Female	B = 2.90, 95% CI (0.42, 6.22), β = 0.17 *p* = 0.097	B = 1.57, 95% CI (-4.36, 7.49), β = 0.09 *p* = 0.64
Education ***(*** *numeric* ***)***	Years of Education		B = 0.35, 95% CI (-0.58, 1.32), β = 0.07 *p* = 0.51
Race/Ethnicity *Ref= Chinese*	Malay	B=-9.61, 95% CI (-16.21, -3.03), β=-0.55 *p* = 0.006**	B=-6.97, 95% CI (-16.65, 2.97), β=-0.41 *p* = 0.21
	Indian	B = 2.88, 95% CI (-1.82, 7.60), β = 0.17 *p* = 0.24	B = 13.22, 95% CI (-1.24, 27.67), β = 0.77 *p* = 0.11
	Other	B = 2.30, 95% CI (-5.06, 9.67), β = 0.13 *p* = 0.55	B = 2.40, 95% CI (-14.99, 10.13),β=-0.14 *p* = 0.73
Psychological Distress *(numeric* ***)***	Change in RSCL Score from Baseline	B = 0.52, 95% CI (0.43, 0.60), β = 0.54 *p* < 0.001***	B = 0.33, 95% CI (0.17, 0.46), β = 0.36 *p* < 0.001***
Emotional Functioning *(numeric)*	Change in Peds QL Score from Baseline		B=-0.19, 95% CI (-0.31, -0.10), β=-0.23 *p* = 0.007**
Cytokine Change From Baseline *(all numeric)*	IL-2		
	IL-4	B=-1.85, 95% CI (-4.76, 1.00), β=-0.06 *p* = 0.22	B=-1.64, 95% CI (-5.74, 2.29), β=-0.06 *p* = 0.45
	IL-6		
	IL-8		
	IL-10	B = 1.87, 95% CI (0.44, 3.30), β = 0.13 *p* = 0.013*	B = 1.52, 95% CI (-0.41, 3.37), β = 0.14β *p* = 0.15
	IFN-ƴ		

From left to right, columns represent broad feature descriptions (Category), specific variables (Variable), regression results for analysis using data from adolescent and young adults cancer patients and healthy controls (AYAC + NC), and regression results using data from adolescent and young adult cancer patients alone (AYAC Only). Each entry represents a regression coefficient (*B*) and corresponding 95% confidence interval ), followed by standardized regression coefficient (β), with associated p-values from Wald-statistics below. For coefficients related to RSCL score change, a positive value indicates that worsening psychological distress (increased RSCL score relative to baseline) is associated with higher fatigue score. For coefficients related to Peds QL score change, a negative value indicates that decreased emotional functioning (decreased Peds QL score relative to baseline) is associated with higher fatigue score. For coefficients related to cytokines, a positive value indicates that an increasing cytokine level (increased measured concentration relative to baseline) is associated with higher fatigue score. Entries left blank indicate a specific variable was not selected with our stepwise feature selection methods

*P < 0.05, **P < 0.01, ***P < 0.001

^NC patients were excluded, changing the reference variable from NC to Active Chemotherapy

^a^R^2^= 0.656 for AYAC and NC

^b^R^2^= 0.591 for AYAC only

For models using AYAC participants alone, all non-cytokine features were selected (Table 4, Supplemental Table 7). However, only baseline fatigue score (Coef(95% CI): 0.77(0.58, 0.95), Std Coef = 0.78, *p* < 0.001, numeric), change in RSCL score (Coef(95% CI): 0.33(0.17, 0.46), Std Coef = 0.36, *p* < 0.001, numeric), and change in PedQL score (Coef(95% CI): -0.19(-0.31, -0.10), Std Coef= -0.23, *p* = 0.007, numeric) were significantly associated with cumulative fatigue score (Table 4, Supplemental Table 7). Chemotherapy status was nearly significant(Coef(95% CI): 3.86(0.29, 7.87), Std Coef= -0.23, *p* = 0.053, ref= Post-Chemotherapy measures), suggesting assessments during active chemotherapy have elevated fatigue scores relative to post-baseline assessments. Amongst cytokines, IL-4 and IL-10 were selected in the final model, with neither achieving statistical significance.

## Discussion

With this longitudinal cohort study, nearly 1 in 3 AYAC participants (~ 30%) experienced clinically important fatigue symptoms over the course of the study, compared to 21% amongst NC. Considering distinct observations, over half (59%) of reported fatigue symptom scores collected for AYAC participants represented clinically important symptoms, with rates above 80% during active chemotherapy. At baseline, AYAC had significantly elevated cytokine levels relative to NC, in addition to poorer symptom scores for several outcomes. Over time, chemotherapy treatment status significantly influenced measured symptomatic scores, with post-chemotherapy measures having less cumulative fatigue symptoms relative to those at baseline and during active chemotherapy. Our modeling efforts further demonstrated significant associations with changing cytokine levels and cumulative fatigue symptoms, highlighting IL-4 and IL-10. Our longitudinal evaluation of the role of cytokines in CRF makes our study unique, especially compared to existing work that merely evaluating patient reported CRF symptoms in the literature [30].

Our study demonstrates that treatment trajectory plays a significant role in measured fatigue symptoms. Other literature reports similar findings, where worsened CRF in AYAC patients was observed 4 months post-baseline in patients receiving treatment [31]. Systematic reviews have highlighted this trend, stating significant increases in fatigue occur in AYAC patients receiving active chemotherapy as opposed to healthy controls [30]. We also found that fatigue scores were elevated at baseline relative to post-treatment measures, suggesting more complex trajectories of fatigue symptoms similar to what is observed in other literature [32]. Such observation aligns with other literature that even amongst a younger patient population, pre-existing conditions like childhood adversity and sleep disturbances could influence a patient’s symptom trajectory [32]. We have incorporated a NC group in this study, in order to account for the background variation of PRO scores. Unexpectedly, we did observe a significant increase in fatigue scores over time amongst this group. Such an increase can be explained by other conditions that non-cancer controls may possess, as well as possible acute infections including COVID-19. This result suggests development of refined measurement tools specific to CRF, less confounded by general illness or fatigue symptoms, could also mitigate the confounding of different symptoms and refine diagnosis in the future. Nonetheless, studies emphasizing longitudinal evaluations of fatigue symptoms in cancer patients also support our findings, demonstrating that cancer patients receiving active chemotherapy have a significant increase in fatigue symptoms relative to prior measurements [33]. Our data highlights that measured CRF symptoms appear to peak with initial cancer diagnosis and during active chemotherapy, suggesting a critical window where interventions can be implemented.

Recommended interventions for CRF require routine visits with various professionals, as no effective pharmaceutical interventions have consensus recommendations in preventing CRF [34, 35]. Implementation of routine exercise regimens, cognitive behavioral therapy, mindfulness-based programs, psychoeducation, and various integrative oncology approaches (i.e. yoga, acupuncture) have had positive impacts in various studies [35]. At this time, there are limited trials evaluating these interventions in AYAC patient populations, as this population is uniquely challenging to engage in such trials [36–38]. Given how common CRF is, creating education programs and opportunities for management in the hospital setting during cancer treatments might be a promising option to mitigate and prevent these symptoms [39, 40].

Our findings highlight a growing body of literature demonstrating that significant relationship between experienced fatigue symptoms and psychological distress [41]. The persistent lack of energy due to CRF is highly distressing to cancer patients, causing a loss of emotional control and self-worth [42]. Distress also causes CRF through a combination of distress-induced insomnia and shared biological pathways including circadian rhythm disruption, serotonin and cytokine dysregulation, as well as HPA axis dysfunction [43, 44]. For AYAC, the co-occurrence of these symptoms reduces not only quality of life but also lifetime productivity by hindering work performance and progression into higher education [45–47] As the population of AYAC continues to grow, it is imperative to ensure timely management of their symptoms and provide comprehensive support during their reintegration into educational and occupational settings [48, 49] This group presents unique challenges, including high levels of unmet psychosocial needs and limited research participation, often due to competing developmental and life responsibilities [50].

We have also evaluated biological factors that are associated with CRF in AYAC. Previous literature has shown that persistent inflammation and counter-regulatory mechanisms, such as IL-10 upregulation, may contribute to prolonged CRF and reinforce its role as a biomarker for CRF [51, 52]. Additional studies have shown significant IL-10 elevation in patients actively receiving chemotherapy, in response to non-specific cytotoxic effects of these treatments [53]. Damaged tissues trigger cytokine signaling cascades, which leads to broader increases in inflammation which mediate observed toxicities from treatment [53]. IL-10 is conventionally viewed as protective against excessive inflammation; its elevated presence in fatigued cancer patients may signal ongoing immune dysregulation. Understanding IL-10’s precise role in CRF could open new avenues for targeted interventions, such as anti-inflammatory therapies or immune-modulating strategies. Whether IL-10 is a proxy for cumulative inflammation or whether its specific action plays a meaningful role in fatigue symptoms needs further investigation. Given the subjective nature of fatigue diagnoses, biomarkers may allow for identification of fatigue induced by cancer and cancer treatment. Elevations in IL-10 can further be utilized as a potential risk factor, allowing for early interventions and prevention of symptoms.

Given the limited knowledge on CRF development and progression, especially among AYAC, identification of key risk factors within our study is important. Furthermore, our inclusion of age-matched NC enhanced the internal validity of the study, minimizing age-related bias and providing a valuable reference for transient biomarker shifts versus clinically relevant changes specific to cancer. Future targeted studies can further evaluate psychological and biomarker level changes to manage and mitigate CRF symptoms unique to AYAC. While this study demonstrates the significant impact of active chemotherapy treatment on symptomatic burden and inflammatory markers, future studies can emphasize data collection at specific time intervals post-treatment to highlight physiological changes associated with longer-term symptom trajectories. Our longitudinal study design demonstrates the value of biomarker and clinical assessment data over time, and how such studies can provide actionable insights that can improve future supportive cancer care and cancer survivorship.

## Study limitations

It is important to note that the interpretability of our findings may be limited by the participants’ clinical heterogeneity, particularly in primary cancer types. Many of the clinical measures utilized were initially created for specific patient populations; Extrapolating their use to NC controls or even in cancer patients aged beyond adolescence allowed for cumulative comparisons amongst all study participants. For instance, the PedsQL survey was validated among AYAC patients aged 25 years old or less [54], with feasibility among adult cancer survivors evaluated only among those who were more than 2 years after treatment [55]. Generalization of our results to a general cancer patient population faces limitations with our AYAC patient cohort. Identifying changes in fatigue and clinically important onset of symptoms relative to our designated baseline level also has limitations, as patients can often experience significant symptoms prior to treatment. This secondary analysis was dependent on complete reporting of biomarkers and symptom assessments; Due to challenges with biospecimen collection amongst this patient population, we were only able to include 50 of 75 patients initially recruited. Lastly, given the exploratory nature of this study, statistical corrections for significance thresholds were not implemented for multiple testings. However, future studies should incorporate multiple testing adjustments to confirm the findings of this study.

## Conclusion

CRF is a highly prevalent burden occurring among AYAC receiving chemotherapy, with 1 out of 4 patients experiencing clinically important CRF along the treatment continuum. Multiple factors can contribute to the onset of CRF among AYAC, which include various demographic, psychological, and biomarker factors. Identification of such factors, as early as diagnosis of malignancy in AYAC, would allow timely and proactive monitoring and management of CRF among AYAC.

## Supplementary Information

Supplementary Material 1.

## Data Availability

The datasets generated during and/or analyzed during the current study are available from the corresponding author on reasonable request.

## Abbreviations

RSCL: Rotterdam Symptom Checklist
CRF: Cancer Related Fatigue
AYAC: Adolescent and Young Adult Cancer Patients
CRF: Non-Cancer Related Fatigue
MFSI-SF: Multidimensional Fatigue Symptom Inventory-Short Form
ACTS: Adolescent and Young Adult Cancer Patients: Cognitive Toxicity on Survivorship
IL: Interleukin
IFN: Interferon
TNF: Tumor Necrosis Factor
PedsQL: Pediatric Quality of Life Inventory
NC: Non-cancer controls
IFN-ƴ: Interferon gamma
TNF-α: Tumor-necrosis factor alpha
SD: Standard deviation
β: Beta coefficient value
