# Supplementary material for: Trajectory and predictors of cancer- and treatment-related fatigue (CRF) in adolescent and young adult cancer patients: a prospective, longitudinal study
Source: BMC Cancer. 2026 Jun 16;26:1019. doi: 10.1186/s12885-026-16324-4 (PMC13501642; doi:10.1186/s12885-026-16324-4)
Supplement: Supplementary file 1 [file 12885_2026_16324_MOESM1_ESM.docx]

**Supplementary Materials**

**Supplementary Methods**

**SM.1 Rotterdam Symptom Checklist (RSCL)**

The RSCL is designed to assess symptom burden in cancer patients. Items on a four-point Likert scale encompass four subscales: physical distress (23 items), psychological distress (7 items), activity level (8 items), and overall valuation of life (1 item). Summing the psychological distress subscale items provides a score (7-28), with higher scores indicating greater psychological distress.

**SM.2 Pediatric Quality of Life Inventory (PedsQL)**

The PedsQL is an assessment designed to evaluate the health-related quality of life in children and adolescents. Using a five-point Likert scale, the assessment includes four subscales: physical functioning (8 items), emotional functioning (5 items), social functioning (5 items), and work/school functioning (5 items). Summing the emotional functioning subscale items provides a score (0-20), with lower scores representing greater emotional distress. PedsQL was chosen in view that it could span across the AYA age spectrum. Three version of the generic core scales were used: teens (ages13–18), young adults (18–25), and adults (age over 26).

**Table S1. Study Participation Over Time**

This table describes sampling trends over time among the study population, including both adolescent and young adult cancer patients (AYAC) and non-cancer controls (NC). The first section “Sample Count by Timepoint” details the total number of complete cases collected for each timepoint. Next, “Sample Count by Treatment Status” reports the total number of complete cases collected by treatment status. Lastly, “Cumulative Samples Provided by Participants” details the number participants providing, 2, 3, 4, or 5 total complete cases throughout the study. Each entry represents count data.

|  | **AYAC** | **NC** |
| --- | --- | --- |
| **Sample Count by Timepoint** |  |  |
| *T1* | 50 | 105 |
| *T2* | 39 | - |
| *T3* | 25 | 105 |
| *T4* | 26 | - |
| *T5* | 33 | - |
| *Total* | 173 | 210 |
| **Sample Count by Treatment Status** |  |  |
| *Active chemotherapy* | 35 | - |
| *Post-chemotherapy* | 88 | - |
| **Cumulative Samples Provided by Participants** |  |  |
| *2 total samples* | 12 | 105 |
| *3 total samples* | 14 | - |
| *4 total samples* | 13 | - |
| *5 total samples* | 11 | - |

**Table S2. Clinical Assessment and Biomarker Measures Over Time Amongst Adult and Young Adult Cancer Patients and Non-Cancer Controls**

From Left to right, the first column represents measurement groupings and names, followed columns representing measurements specific to timepoints in the study (T1- T5). The first grouping of columns represent entries related to samples for adult and young adult cancer patients, followed by entries for non-cancer controls. For clinical measures, each entry represents mean and standard deviation (SD) for a given measure and timepoint (mean (SD)). For Cytokines, each entry represents median and interquartile range (IQR) for a given cytokine level and timepoint (median (IQR)).

|  | | **Adult and Young Adult Cancer Patients**  50 Unique Participants  173 Total samples | | | | | **Non-Cancer Controls**  105 Unique Participants  210 Total Samples | |
| --- | --- | --- | --- | --- | --- | --- | --- | --- |
|  | | T1  (baseline) | T2 | T3 | T4 | T5 | T1  (baseline) | T3 |
| **^a^MFSI-SF**  *mean (SD)* | |  |  |  |  |  |  |  |
| General  Subscale | | 5.3 (4.1) | 6.4 (4.6) | 4.1 (4.0) | 3.9 (4.3) | 3.3 (3.3) | 4.8 (4.5) | 5.4 (4.6) |
| Physical  Subscale | | 3.1 (3.6) | 5.1 (4.3) | 3.8 (3.7) | 3.2 (3.7) | 2.2 (3.0) | 2.2 (3.0) | 2.8 (3.6) |
| Emotional  Subscale | | 6.5 (4.5) | 5.1 (4.0) | 4.9 (4.8) | 3.2 (3.6) | 3.0 (3.3) | 3.1 (3.5) | 4.1 (4.4) |
| Mental  Subscale | | 2.8 (3.6) | 3.9 (3.6) | 2.7 (3.0) | 3.5 (4.0) | 2.4 (2.9) | 3.1 (3.5) | 3.5 (3.8) |
| Vigor  Subscale | | 13.3 (4.7) | 13.6 (5.6) | 15.2 (5.6) | 16.8 (4.9) | 15.2 (6.8) | 15.1 (5.0) | 14.1 (5.3) |
| Total Score | | 4.4 (16.4) | 6.9 (17.4) | 0.4 (17.4) | -2.9 (17.0) | -4.3 (15.0) | -1.9 (15.6) | 1.6 (17.7) |
| **^b^RSCL**  *mean (SD)* | |  |  |  |  |  |  |  |
| Physical Symptom Distress | | 12.8 (12.2) | 21.3 (12.1) | 12.1 (8.9) | 10.4 (10.4) | 10.6 (12.2) | 8.3 (7.4) | 9.1 (9.1) |
| Psychological  Distress | | 28.5 (19.1) | 24.8 (17.3) | 20.4 (18.7) | 18.1 (17.6) | 15.7 (13.8) | 16.3 (15.3) | 18.0 (19.1) |
| Activity Level | | 4.7 (7.8) | 5.1 (8.3) | 2.9 (6.2) | 1.9 (5.5) | 1.0 (4.0) | 0.06 (0.4) | 0.03 (0.3) |
| Overall Valuation  of Life | | 23.6 (18.5) | 27.6 (14.6) | 18.7 (14.7) | 13.5 (12.5) | 15.2 (14.0) | 16.0 (16.2) | 16.3 (17.1) |
| **^c^PedsQL**  *mean (SD)* | |  |  |  |  |  |  |  |
| Physical  Functioning | | 79.9 (20.4) | 67.7 (20.0) | 80.8 (19.8) | 86.4 (15.4) | 88.4 (15.6) | 92.3 (11.5) | 92.9 (10.8) |
| Emotional  Functioning | | 66.2 (20.4) | 67.7 (17.2) | 74.2 (22.2) | 78.8 (20.6) | 82.4 (17.9) | 78.9 (19.5) | 79.1 (20.3) |
| Social  Functioning | | 90.3 (14.0) | 88.8 (13.3) | 91.0 (13.2) | 91.5 (14.1) | 95.8 (11.7) | 91.2 (12.4) | 91.2 (12.4) |
| Work/School Functioning | | 79.6 (18.1) | 65.9 (21.8) | 82.1 (19.4) | 85.2 (15.2) | 87.5 (14.5) | 85.6 (15.0) | 85.6 (15.5) |
|  | **Cytokines**  *median (IQR)* |  |  |  |  |  |  |  |
|  | IL-2 (pg/mL) | 0.0 (0.0, 1.3) | 0.3 (0.0, 1.7) | 0.0 (0.0, 1.3) | 0.0 (0.0, 1.0) | 0.0 (0.0, 0.7) | 0.0 (0.0, 0.0) | 0.0 (0.0, 0.5) |
|  | IL-4 (pg/mL) | 0.0 (0.0, 0.6) | 0.1 (0.0, 1.1) | 0.0 (0.0, 1.1) | 0.0 (0.0, 0.1) | 0.0 (0.0, 0.6) | 0.0 (0.0, 0.0) | 0.0 (0.0, 0.0) |
|  | IL-6 (pg/mL) | 2.0 (1.0, 3.3) | 1.8 (1.0, 3.2) | 1.5 (1.1, 2.9) | 1.3 (0.7, 2.4) | 1.3 (0.7, 2.0) | 0.5 (0.0, 1.0) | 0.5 (0.1, 1.0) |
|  | IL-8 (pg/mL) | 5.5 (3.5, 11.9) | 6.8 (4.9, 11.8) | 6.4 (4.0, 9.5) | 4.7 (3.4, 7.3) | 4.6 (3.4, 7.2) | 4.3 (2.5, 5.6) | 4.1 (2.3, 5.2) |
|  | IL-10 (pg/mL) | 0.5 (0.0, 1.6) | 0.4 (0.0, 1.6) | 0.5 (0.0, 2.1) | 0.5 (0.0, 1.2) | 0.7 (0.0, 1.1) | 0.0 (0.0, 0.5) | 0.0 (0.0, 0.0) |
|  | IFN-ƴ (pg/mL) | 0.8 (0.3, 1.6) | 1.0 (0.3, 2.0) | 0.7 (0.0, 1.8) | 0.5 (0.2, 1.2) | 0.5 (0.2, 1.2) | 0.3 (0.0, 0.6) | 0.0 (0.0, 0.5) |
|  | TNF-α (pg/mL) | 9.7 (6.2, 15.7) | 11.1 (7.2, 18.6) | 8.9 (6.5, 11.4) | 11.0 (7.8, 13.8) | 9.8 (7.4, 12.1) | 9.2 (6.5, 12.0) | 8.8 (5.3, 11.8) |

**Table S3. Clinical Assessment and Biomarker Measures by Treatment Status**

From Left to right, the first column represents measurement groupings and names, followed columns representing measurements specific to treatment status including samples taken during active chemotherapy, samples taken post-chemotherapy, and samples for non-cancer cancer controls post-baseline. For clinical measures, each entry represents mean and standard deviation (SD) for a given measure (mean (SD)). For Cytokines, each entry represents median and interquartile range (IQR) for a given cytokine and timepoint (median (IQR)).

|  | | Active  Chemotherapy  (35 samples) | Post-Chemotherapy  (88 samples) | Non-Cancer Controls  (105 samples) |
| --- | --- | --- | --- | --- |
| **^a^MFSI-SF**  *mean (SD)* | |  |  |  |
| General  Subscale | | 6.5 (4.4) | 3.8 (3.9) | 5.1 (4.5) |
| Physical  Subscale | | 5.6 (4.2) | 2.9 (3.5) | 2.5 (3.3) |
| Emotional  Subscale | | 5.5 (4.0) | 3.6 (3.9) | 3.6 (4.0) |
| Mental  Subscale | | 4.1 (3.7) | 2.8 (3.3) | 3.3 (3.6) |
| Vigor  Subscale | | 13.8 (5.6) | 15.5 (6.0) | 14.6 (5.2) |
| Total Score | | 7.9 (18.0) | -2.4 (16.0) | -0.1 (16.7) |
| **^b^RSCL**  *mean (SD)* | |  |  |  |
| Physical Symptom Distress | | 20.6 (12.2) | 11.7 (11.1) | 8.7 (8.3) |
| Psychological  Distress | | 25.0 (16.4) | 18.1 (16.9) | 17.1 (17.3) |
| Activity Level | | 5.8 (8.7) | 1.7 (5.0) | 0.04 (0.4) |
| Overall Valuation  of Life | | 26.5 (16.0) | 16.7 (13.8) | 16.2 (16.6) |
| **^c^PedsQL**  *mean (SD)* | |  |  |  |
| Physical  Functioning | | 66.2 (19.9) | 85.3 (16.9) | 92.6 (11.1) |
| Emotional  Functioning | | 65.9 (17.4) | 79.1 (19.6) | 79.0 (19.9) |
| Social  Functioning | | 87.3 (14.9) | 93.5 (12.0) | 91.2 (12.4) |
| Work/School Functioning | | 65.6 (21.8) | 84.2 (17.1) | 85.6 (15.2) |
|  | **Cytokines**  *median (IQR)* |  |  |  |
|  | IL-2 (pg/mL) | 0.3 (0.0, 1.4) | 0.0 (0.0, 1.2) | 0.0 (0.0, 0.3) |
|  | IL-4 (pg/mL) | 0.0 (0.0, 0.9) | 0.0 (0.0, 0.7) | 0.0 (0.0, 0.0) |
|  | IL-6 (pg/mL) | 1.5 (1.0, 2.4) | 1.4 (0.8, 2.9) | 0.5 (0.0, 1.0) |
|  | IL-8 (pg/mL) | 6.6 (4.7, 8.9) | 5.3 (3.6, 8.5) | 4.2 (2.5, 5.4) |
|  | IL-10 (pg/mL) | 0.7 (0.0, 1.4) | 0.5 (0.0, 1.5) | 0.0 (0.0, 0.2) |
|  | IFN-ƴ (pg/mL) | 1.0 (0.2, 1.8) | 0.6 (0.2, 1.6) | 0.2 (0.0, 0.6) |
|  | TNF-α (pg/mL) | 9.7 (8.3, 13.9) | 10.7 (6.8, 15.8) | 8.9 (5.4, 12.0) |

**Table S4: Impact of Treatment Status on Measured Cytokine Levels Based from Preliminary Regression Analysis Amongst Adolescent and Young Adult Cancer Patients and Non-Cancer Controls**

The first column represents different cytokines utilized as outcomes for regression analysis, with subsequent columns representing treatment status (for adolescent and young adult samples (AYAC)) or post-baseline measurements (for non-cancer controls (NC)). Reported results are regression coefficients (B), 95% confidence interval $(CI$), followed by standardized regression coefficients (β), and their corresponding Wald statistic reported below them. AYAC and NC data were utilized in separate groupings with different regressions conducted, Panel **(A)** represents regression results amongst AYAC participants, while panel **(B)** represents regression results for non-cancer controls. Coefficients are reported in reference to baseline measures

(A) AYAC

| Cytokines | $Active$  $Chemotherapy$  Ref= Baseline Measures | $Post-$  Chemotherapy  Ref= Baseline Measures |
| --- | --- | --- |
| IL-2 | B=0.1, 95% CI (-0.3, 0.5), β=0.09  p= 0.57 | B=0.03, 95% CI (-0.3, 0.3), β=0.03  p= 0.83 |
| IL-4 | B=0.1, 95% CI (-0.2, 0.4), β=0.13  p= 0.37 | B=0.05, 95% CI (-0.2, 0.3), β=0.05  p= 0.68 |
| IL-6 | B=0.2, 95% CI (-0.1, 0.4), β=0.23  p= 0.19 | B=-0.04, 95% CI (-0.2, 0.1), β=-0.06  p= 0.64 |
| IL-8 | B=0.06, 95% CI (-0.02, 0.1), β=0.23  p= 0.14 | B=-0.04, 95% CI (-0.1, 0.03), β=-0.14  p= 0.24 |
| IL-10 | B=0.04, 95% CI (-0.3, 0.3), β=0.03  p= 0.80 | B=0.2, 95% CI (-0.1, 0.4), β=0.14  p= 0.20 |
| IFN-ƴ | B=-0.1, 95% CI (-0.3, 0.2), β=-0.09  p= 0.59 | B=-0.2, 95% CI (-0.4, 0.05), β=-0.22  p= 0.12 |
| TNF-α | B=0.1, 95% CI (-0.2, 0.3), β=0.09  p= 0.64 | B=0.01, 95% CI (-0.2, 0.2), β=0.02  p= 0.90 |

(B) NC

| Cytokines | $Post-Baseline$  Ref= Baseline Measures |
| --- | --- |
| IL-2 | B=0.2, 95% CI (-0.1, 0.3), β=0.20  p= 0.0032** |
| IL-4 | B=-0.03, 95% CI (-0.1, 0.05), β=-0.05  p= 0.44 |
| IL-6 | B=-0.01, 95% CI (-0.1, 0.1), β=0.02  p= 0.80 |
| IL-8 | B=-0.02, 95% CI (-0.1, 0.03), β=-0.03  p= 0.45 |
| IL-10 | B=-0.2, 95% CI (-0.3, -0.1), β=-0.26  p= 2.41E-5*** |
| IFN-ƴ | B=-0.2, 95% CI (-0.3, -0.1), β=-0.23  p= 0.00016*** |
| TNF-α | B=-0.1, 95% CI (-0.2, 0.02), β=-0.08  p= 0.12 |

**Table S5: Impact of Treatment Status on Measured Cytokine Levels from Preliminary Regression Analysis Stratified by Cancer Status and Onset of Clinically Important Fatigue Symptoms**

The first column represents different cytokines utilized as outcomes for regression analysis, with subsequent columns representing treatment status (for adolescent and young adult samples (AYAC)) or post-baseline measurements (for non-cancer controls (NC)). Reported results are regression coefficients (B), 95% confidence interval $(CI$), followed by standardized regression coefficients (β), and their corresponding Wald statistic reported below them. Four distinct groupings were utilized to conduct separate regression analyses including AYAC participants that experienced clinically important fatigue symptoms (panel A, AYAC + Clinically Important Fatigue), AYAC participants that did not experience clinically important symptoms (panel B, AYAC + No Clinically Important Fatigue), NC participants that experienced clinically important fatigue symptoms (panel C, AYAC + Clinically Important Fatigue), and NC participants that did not experience clinically important symptoms (panel D, AYAC + No Clinically Important Fatigue). Coefficients are reported in reference to baseline measures

(A) AYAC + Clinically Important Fatigue

| Cytokines | Active Chemotherapy  *Ref= Baseline Measures* | $Post$*-Chemotherapy*  *Ref= Baseline Measures* |
| --- | --- | --- |
| IL-2 (pg/mL) | B=-0.04, 95% CI (-0.7, 0.7), β=-0.03  p= 0.91 | B=-0.07, 95% CI (-0.6, 0.5), β=-0.05  p=0.81 |
| IL-4 (pg/mL) | B=-0.04, 95% CI (-0.6, 0.6), β=-0.03  p= 0.90 | B=-0.05, 95% CI (-0.5, 0.4), β=-0.04  p=0.84 |
| IL-6 (pg/mL) | B=0.2, 95% CI (-0.2, 0.6), β=0.37  p= 0.26 | B=-0.1, 95% CI (-0.4, 0.2), β=-0.20  p=0.42 |
| IL-8 (pg/mL) | B=0.1, 95% CI (-0.1, 0.3), β=0.35  p= 0.23 | B=-0.1, 95% CI (-0.3, 0.01), β=-0.40  p=0.080 |
| IL-10 (pg/mL) | B=-0.1, 95% CI (-0.7, 0.5), β=-0.09  p= 0.72 | B=-0.02, 95% CI (-0.5, 0.4), β=-0.02  p=0.92 |
| IFN-ƴ (pg/mL) | B=-0.4, 95% CI (-0.9, 0.1), β=-0.48  p= 0.10 | B=-0.3, 95% CI (-0.7, 0.04), β=-0.39  p=0.092 |
| TNF-α (pg/mL) | B=0.02, 95% CI (-0.4, 0.4), β=0.04  p= 0.91 | B=-0.1, 95% CI (-0.4, 0.2), β=-0.23  p=0.46 |

(B) AYAC + No Onset of Clinically Important Fatigue

| Cytokines | Active Chemotherapy  *Ref= Baseline Measures* | $Post$*- Chemotherapy*  *Ref= Baseline Measures* |
| --- | --- | --- |
| IL-2 (pg/mL) | B=0.2, 95% CI (-0.3, 0.6), β=0.15  p=0.44 | B=0.1, 95% CI (-0.3, 0.4), β=0.06  p=0.72 |
| IL-4 (pg/mL) | B=0.2, 95% CI (-0.1, 0.6), β=0.22  p=0.22 | B=0.1, 95% CI (-0.2, 0.4), β=0.08  p=0.57 |
| IL-6 (pg/mL) | B=0.1, 95% CI (-0.2, 0.4), β=0.15  p=0.46 | B=-0.0003, 95% CI (-0.2, 0.2), β=-4.8E-4  p=1.00 |
| IL-8 (pg/mL) | B=0.05, 95% CI (-0.05, 0.1), β=0.18  p=0.33 | B=-0.001, 95% CI (-0.1, 0.1), β=-5.0E-3  p=0.97 |
| IL-10 (pg/mL) | B=0.1, 95% CI (-0.2, 0.4), β=0.09  p=0.56 | B=0.2, 95% CI (-0.05, 0.5), β=0.21  p=0.11 |
| IFN-ƴ (pg/mL) | B=0.1, 95% CI (-0.3, 0.4), β=0.09  p=0.68 | B=-0.1, 95% CI (-0.4, 0.2), β=-0.14  p=0.44 |
| TNF-α (pg/mL) | B=0.1, 95% CI (-0.3, 0.4), β=0.09  p=0.72 | B=0.1, 95% CI (-0.2, 0.4), β=0.08  p=0.67 |

(C) NC + Onset of Clinically Significant Fatigue

| Cytokines | $Post-Basline$  Ref= *Baseline Measures* |
| --- | --- |
| IL-2 (pg/mL) | B=-0.05, 95% CI (-0.3, 0.2), β=-0.07  p=0.67 |
| IL-4 (pg/mL) | B=-0.07, 95% CI (-0.2, 0.9), β=-0.13  p=0.41 |
| IL-6 (pg/mL) | B=0.1, 95% CI (-0.1, 0.3), β=0.15  p=0.27 |
| IL-8 (pg/mL) | B=0.04, 95% CI (-0.003, 0.08), β=0.17  p=0.079 |
| IL-10 (pg/mL) | B=-0.2, 95% CI (-0.4, -0.1), β=-0.35  p=0.023* |
| IFN-ƴ (pg/mL) | B=0.02, 95% CI (-0.2, 0.2), β=-0.03  p=0.84 |
| TNF-α (pg/mL) | B=-0.2, 95% CI (-0.5, 0.06), β=-0.22  p=0.13 |

(D) NC + No Onset of Clinically Significant Fatigue

| Cytokines | $Post-Basline$  Ref= *Baseline Measures* |
| --- | --- |
| IL-2 (pg/mL) | B=0.2, 95% CI (0.1, 0.4), β=0.27  p=0.00056*** |
| IL-4 (pg/mL) | B=-0.02, 95% CI (-0.1, 0.1), β=-0.03  p=0.64 |
| IL-6 (pg/mL) | B=-0.02, 95% CI (-0.1, 0.1), β=-0.02  p=0.77 |
| IL-8 (pg/mL) | B=-0.03, 95% CI (-0.1, 0.03), β=-0.06  p=0.28 |
| IL-10 (pg/mL) | B=-0.2, 95% CI (-0.3, -0.1), β=-0.25  p=0.00032*** |
| IFN-ƴ (pg/mL) | B=-0.2, 95% CI (-0.4, -0.1), β=-0.28  p=3.64E-5*** |
| TNF-α (pg/mL) | B=-0.04, 95% CI (-0.1, 0.1), β=-0.04  p=0.45 |

**Table S6. Model Diagnostics for Step-wise Model Building Exercise Predicting Cumulative Fatigue Score Using Data from All Study Participants**

From Left to right, the first column represents the stage of model building represented, followed by a description of the variables considered for that stage, followed by numbered models. Next, specific models conducted along with corresponding Wald statistics for variables included in each model, followed by AIC scores for each model ran. The first model occurring within each stage includes all variables relevant to that stage, followed by subsequent removal of different features in models reported below it. Models selected achieving lowest AIC values, in addition to selected features, are boldened for ease of reference.

| Stage | Description | Model # | Model | AIC |
| --- | --- | --- | --- | --- |
| 1 | Baseline  **chemo group (ref: Active Chemotherapy),**  **Baseline Fatigue Score** | **1** | **Chemo Group (Active Chemo: P=0.71, Post-Chemo: P=0.014), Baseline MFSI-SF Total Score (P<0.001)** | **1814.7** |
| 2 | Demographics  Age,  **gender (ref: male),** **ethnicity (ref: Chinese),**  Sex (ref= Male), | 2 | Chemo Group (Active Chemo: P=0.69, Post-Chemo: P=0.054), Baseline MFSI-SF Total Score (P<0.001), Age (P=0.69), Gender (P=0.054), Years of Education (P=0.94), Ethnicity (Malay: P=0.092, Indian: P=0.14, Others: P=0.67) | 1802.4 |
|  | Years of Education | 3 | Chemo Group (Active Chemo: P=0.68, Post-Chemo: P=0.011), Baseline MFSI-SF Total Score (P<0.001), Age (P=0.70), Gender (P=0.052), Ethnicity (Malay: P=0.086, Indian: P=0.13, Others: P=0.67) | 1800.1 |
|  |  | **4** | **Chemo Group (Active Chemo: P=0.63, Post-Chemo: P=0.012), Baseline MFSI-SF Total Score (P<0.001), Gender (P=0.054), Ethnicity (Malay: P=0.068, Indian: P=0.14, Others: P=0.66)** | **1796.9** |
|  |  | 5 | Chemo Group (Active Chemo: P=0.89, Post-Chemo: P=0.0019), Baseline MFSI-SF Total Score (P<0.001), Gender (P=0.11) | 1810.8 |
| 3 | Psychological Factors Peds QL,  **RSCL** | 6 | Chemo Group (Active Chemo: P=0.094, Post-Chemo: P=0.95), Baseline MFSI-SF Total Score (P<0.001), Gender (P=0.16), Ethnicity (Malay: P=0.016, Indian: P=0.26, Others: P=0.47), RSCL Psychological Distress (P<0.001), PedsQL Emotional Functioning (P=0.029) | 1695.9 |
|  |  | **7** | **Chemo Group (Active Chemo: P=0.070, Post-Chemo: P=0.92), Baseline MFSI-SF Total Score (P<0.001), Gender (P=0.14), Ethnicity (Malay: P=0.012, Indian: P=0.21, Others: P=0.46), RSCL Psychological Distress (P<0.001)** | **1694.4** |
| 4 | **Cytokines**  IL-2,  **IL-4,**  IL-6,  IL-8,  **IL-10,** | 8 | Chemo Group (Active Chemo: P=0.087, Post-Chemo: P=1.0), Baseline MFSI-SF Total Score (P<0.001), Gender (P=0.11), Ethnicity (Malay: P=0.0082, Indian: P=0.25, Others: P=0.58), RSCL Psychological Distress (P<0.001), IL-2 (P=0.83), IL-4 (P=0.19), IL-6 (P=0.97), IL-8 (P=0.54), IL-10 (P=0.038), IFN-ƴ (P=0.44), TNF-α (P=0.90) | 1700.2 |
|  | IL-10,  IFN-ƴ,  TNF-α | 9 | Chemo Group (Active Chemo: P=0.084, Post-Chemo: P=0.99), Baseline MFSI-SF Total Score (P<0.001), Gender (P=0.11), Ethnicity (Malay: P=0.0081, Indian: P=0.25, Others: P=0.58), RSCL Psychological Distress (P<0.001), IL-2 (P=0.83), IL-4 (P=0.19), IL-8 (P=0.53), IL-10 (P=0.036), IFN-ƴ (P=0.45), TNF-α (P=0.90) | 1697.3 |
|  |  | 10 | Chemo Group (Active Chemo: P=0.084, Post-Chemo: P=1.0), Baseline MFSI-SF Total Score (P<0.001), Gender (P=0.11), Ethnicity (Malay: P=0.0078, Indian: P=0.25, Others: P=0.57), RSCL Psychological Distress (P<0.001), IL-2 (P=0.83), IL-4 (P=0.17), IL-8 (P=0.54), IL-10 (P=0.035), IFN-ƴ (P=0.44) | 1693.2 |
|  |  | 11 | Chemo Group (Active Chemo: P=0.074, Post-Chemo: P=0.96), Baseline MFSI-SF Total Score (P<0.001), Gender (P=0.11), Ethnicity (Malay: P=0.0072, Indian: P=0.25, Others: P=0.56), RSCL Psychological Distress (P<0.001), IL-4 (P=0.13), IL-8 (P=0.55), IL-10 (P=0.020), IFN-ƴ (P=0.42) | 1692.4 |
|  |  | 12 | Chemo Group (Active Chemo: P=0.070, Post-Chemo: P=1.0), Baseline MFSI-SF Total Score (P<0.001), Gender (P=0.092), Ethnicity (Malay: P=0.0063, Indian: P=0.24, Others: P=0.55), RSCL Psychological Distress (P<0.001), IL-4 (P=0.16), IL-10 (P=0.015), IFN-ƴ (P=0.34) | 1689.9 |
|  |  | **13** | **Chemo Group (Active Chemo: P=0.049, Post-Chemo: P=0.99), Baseline MFSI-SF Total Score (P<0.001), Gender (P=0.097), Race/Ethnicity (Malay: P=0.0060, Indian: P=0.24, Others: P=0.55), RSCL Psychological Distress (P<0.001), IL-4 (P=0.22), IL-10 (P=0.013)** | **1688.6** |
|  |  | 14 | Chemo Group (Active Chemo: P=0.066, Post-Chemo: P=0.93), Baseline MFSI-SF Total Score (P<0.001), Gender (P=0.088), Ethnicity (Malay: P=0.0060, Indian: P=0.26, Others: P=0.59), RSCL Psychological Distress (P<0.001), IL-10 (P=0.030) | 1690.8 |

Abbreviations: AIC, Akaike Information Criterion; IFN-ƴ, interferon-gamma; IL, interleukin; MFSI-SF, Multidimensional Fatigue Symptom Inventory – Short Form; NC, non-cancer control; PedsQL, Pediatric Quality of Life Inventory; RSCL, Rotterdam Symptom Checklist; TNF-α, tumor necrosis factor-alpha

**Table S7. Model Diagnostics for Step-wise Model Building Exercise Predicting Cumulative Fatigue Score Using Data from Adolescent and Young Adult Cancer Patients Only**

From Left to right, the first column represents the stage of model building represented, followed by a description of the variables considered for that stage, followed by numbered models. Next, specific models conducted along with corresponding Wald statistics for variables included in each model, followed by AIC scores for each model ran. The first model occurring within each stage includes all variables relevant to that stage, followed by subsequent removal of different features in models reported below it. Models selected achieving lowest AIC values are boldened, in addition to features selected, for ease of reference.

| Stage | Description | Model # | Model | AIC |
| --- | --- | --- | --- | --- |
| 1 | Baseline  **chemo group (ref: NC),**  **Baseline Fatigue Score** | **1** | **Chemo Group (Active Chemo: P=0.0027), Baseline MFSI-SF Total Score (P<0.001)** | **971.5** |
| 2 | Demographics  **Age**  **gender (ref: male) ethnicity (ref: Chinese)** | **2** | **Chemo Group (Active Chemo: P<0.001), Baseline MFSI-SF Total Score (P<0.001), Age (P=0.42), Gender (P=0.23), Years of Education (P=0.070), Ethnicity (Malay: P=0.72, Indian: P=0.11, Others: P=0.46)** | **951.9** |
|  | **Years of Education** | 3 | Chemo Group (Active Chemo: P=0.0012), Baseline MFSI-SF Total Score (P<0.001), Age (P=0.34), Gender (P=0.12), Years of Education (P=0.067) | 966.9 |
|  |  | 4 | Chemo Group (Active Chemo: P=0.0013), Baseline MFSI-SF Total Score (P<0.001), Gender (P=0.12), Years of Education (P=0.10) | 965.2 |
|  |  | 5 | Chemo Group (Active Chemo: P=0.0014), Baseline MFSI-SF Total Score (P<0.001), Years of Education (P=0.079) | 970.0 |
| 3 | Psychological Factors  **Peds QL,**  **RSCL** | **6** | **Chemo Group (Active Chemo: P=0.055), Baseline MFSI-SF Total Score (P<0.001), Age (P=0.36), Gender (P=0.91), Years of Education (P=0.29), Ethnicity (Malay: P=0.36, Indian: P=0.049, Others: P=0.76), RSCL Psychological Distress (P<0.001), PedsQL Emotional Functioning (P=0.0060)** | **922.1** |
|  |  | **7** | Chemo Group (Active Chemo: P=0.014), Baseline MFSI-SF Total Score (P<0.001), Age (P=0.66), Gender (P=0.73), Years of Education (P=0.31), Ethnicity (Malay: P=0.30, Indian: P=0.064, Others: P=0.73), RSCL Psychological Distress (P<0.001) | 924.2 |
| 4 | Cytokines  IL-2,  **IL-4,**  IL-6,  IL-8,  **IL-10,**  IFN-ƴ, | 8 | Chemo Group (Active Chemo: P=0.067), Baseline MFSI-SF Total Score (P<0.001), Age (P=0.54), Gender (P=0.55), Years of Education (P=0.58), Ethnicity (Malay: P=0.20, Indian: P=0.11, Others: P=0.76), RSCL Psychological Distress (P<0.001), PedsQL Emotional Functioning (P=0.0093), IL-2 (P=0.96), IL-4 (P=0.64), IL-6 (P=0.88), IL-8 (P=0.66), IL-10 (P=0.23), IFN-ƴ (P=0.67), TNF-α (P=0.76) | 929.3 |
|  | TNF-α | 9 | Chemo Group (Active Chemo: P=0.066), Baseline MFSI-SF Total Score (P<0.001), Age (P=0.53), Gender (P=0.55), Years of Education (P=0.58), Ethnicity (Malay: P=0.19, Indian: P=0.11, Others: P=0.76), RSCL Psychological Distress (P<0.001), PedsQL Emotional Functioning (P=0.0089), IL-4 (P=0.60), IL-6 (P=0.88), IL-8 (P=0.65), IL-10 (P=0.15), IFN-ƴ (P=0.65), TNF-α (P=0.76) | 929.4 |
|  |  | 10 | Chemo Group (Active Chemo: P=0.060), Baseline MFSI-SF Total Score (P<0.001), Age (P=0.53), Gender (P=0.55), Years of Education (P=0.58), Ethnicity (Malay: P=0.19, Indian: P=0.11, Others: P=0.76), RSCL Psychological Distress (P<0.001), PedsQL Emotional Functioning (P=0.0081), IL-4 (P=0.59), IL-8 (P=0.66), IL-10 (P=0.13), IFN-ƴ (P=0.66), TNF-α (P=0.77) | 926.7 |
|  |  | 11 | Chemo Group (Active Chemo: P=0.060), Baseline MFSI-SF Total Score (P<0.001), Age (P=0.54), Gender (P=0.57), Years of Education (P=0.54), Ethnicity (Malay: P=0.19, Indian: P=0.11, Others: P=0.77), RSCL Psychological Distress (P<0.001), PedsQL Emotional Functioning (P=0.0074), IL-4 (P=0.55), IL-8 (P=0.59), IL-10 (P=0.13), IFN-ƴ (P=0.63) | 923.2 |
|  |  | 12 | Chemo Group (Active Chemo: P=0.048), Baseline MFSI-SF Total Score (P<0.001), Age (P=0.53), Gender (P=0.59), Years of Education (P=0.52), Ethnicity (Malay: P=0.20, Indian: P=0.11, Others: P=0.77), RSCL Psychological Distress (P<0.001), PedsQL Emotional Functioning (P=0.0068), IL-4 (P=0.57), IL-8 (P=0.67), IL-10 (P=0.13) | 921.4 |
|  |  | **13** | **Chemo Group (Active Chemo: P=0.053), Baseline MFSI-SF Total Score (P<0.001), Age (P=0.52), Gender (P=0.64), Years of Education (P=0.51), Ethnicity (Malay: P=0.21, Indian: P=0.11, Others: P=0.73), RSCL Psychological Distress (P<0.001), PedsQL Emotional Functioning (P=0.0072), IL-4 (P=0.45), IL-10 (P=0.15)** | **919.1** |
|  |  | 14 | Chemo Group (Active Chemo: P=0.061), Baseline MFSI-SF Total Score (P<0.001), Age (P=0.43), Gender (P=0.68), Years of Education (P=0.48), Ethnicity (Malay: P=0.22, Indian: P=0.11, Others: P=0.68), RSCL Psychological Distress (P<0.001), PedsQL Emotional Functioning (P=0.0074), IL-10 (P=0.21) | 921.1 |

Abbreviations: AIC, Akaike Information Criterion; AYAC, adolescent and young adult cancer; IFN-ƴ, interferon-gamma; IL, interleukin; MFSI-SF, Multidimensional Fatigue Symptom Inventory – Short Form; PedsQL, Pediatric Quality of Life Inventory; RSCL, Rotterdam Symptom Checklist; TNF-α, tumor necrosis factor-alpha
